# 

**Published:** 1890-11-29

**Authors:** 


PRICE ONE PENNY.

No. 218, Vol. IX.—November 29th, 1890.

[Registered at the General Post Office as a Newspaper for transmission in the United Kingdom and Abroad.]

WITH EXTRA NURSING SUPPLEMENT

Per Annum, 6s. 6d.; post free.

Monthly Parts, 6d.; post free 8d.

Ditto per Annum, 8s., post free.

### THE HOSPITAL

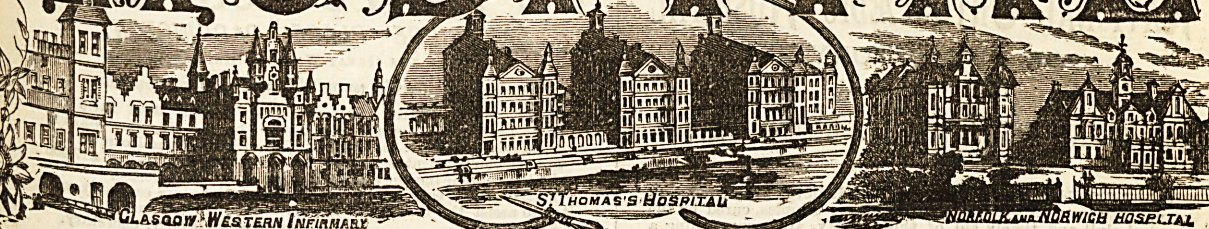

A WEEKLY JOURNAL OF

Science, Medicine, Nursing and Philanthropy.

SATURDAY, NOVEMBER 29th, 1890.

Twenty-One or Twenty-Three? - 133
Passing Topics.—The National Pension Fund and Workers.—II. - 134
Nurses' Food, Work, and Hours of Recreation - 135
Editor's Letter Box—Diphtheria at Marlborough College - 137
Turning Over New Leaves.—Captain Cook - 138
The Doctor's Armchair.—Koch's Notes of Warning - 139
Notes and News - 140
Annotations.—Lady Rosebery—A Word to the Governors of the London Hospital—Diphtheria at Croydon - 142
Scraps and Gleanings - 147
The Practitioner's Mirror and Retrospect:—
The Post-Graduate Course at the Brompton Consumption Hospital.—Pulmonary Phthisis - 143
The London Post-Graduate Course at the Paddington Infirmary.—Rheumatism, 144; Pathology - 145
Surgery.—A New Antiseptic Treatment of Wounds - 146
New Drugs, Appliances, and Things Medical.—Tea, Coffee, or Cocoa? 146; "Cherry Blossom" Soap - 147
The Student of Medicine.—Editorial Notes—Appointments—Students' Medical Societies—Notes from the Schools—Pass Lists—Athletics—Events for the Month of December - 148

### EXTRA SUPPLEMENT—THE NURSING MIRROR.

En Passant.—Religion at Leicester—Short Items—A Cornish Story—Another Hostel—Nurses' Missionary Association - xli

Lectures on Surgical Ward Work and Nursing. By Alex. Miles, M.B. (Edin.), C.M., F.R.C.S.E.—Lecture V.—Wool and Lint - xlii

The Glasgow Sick Poor and Private Nursing Institution - xlii

Death in Our Ranks - xlii

The Trained Nurse in the Nursery - xliii

Nurses on Their Travels.—Notes from Australia - xliv

The Institute of Medical Electricity - xliv

Everybody's Opinion.—Tetanus, xliv; Ladies on Committees—Shall Women Propose?—Male Nurses - xlv

Notes and Queries—Vacancies - xlv

Our Understandings (*conclusion*) - xlvi

Hospital Memories - xlvi

Amusements and Relaxation - xlvi

